# Predicting Increased Blood Pressure Using Machine Learning

**DOI:** 10.1155/2014/637635

**Published:** 2014-01-23

**Authors:** Hudson Fernandes Golino, Liliany Souza de Brito Amaral, Stenio Fernando Pimentel Duarte, Cristiano Mauro Assis Gomes, Telma de Jesus Soares, Luciana Araujo dos Reis, Joselito Santos

**Affiliations:** ^1^Laboratório de Investigação da Arquitetura Cognitiva, Universidade Federal de Minas Gerais, 30000-000 Belo Horizonte, Minas Gerais, MG, Brazil; ^2^Instituto Multidisciplinar de Saúde, Universidade Federal da Bahia, 40000-000 Bahia, BA, Brazil; ^3^Núcleo de Pós-Graduação, Pesquisa e Extenção, Faculdade Independente do Nordeste, São Luís Avenue, 1305, 45000-000 Candeias, Vitória da Conquista, BA, Brazil

## Abstract

The present study investigates the prediction of increased blood pressure by body mass index (BMI), waist (WC) and hip circumference (HC), and waist hip ratio (WHR) using a machine learning technique named classification tree. Data were collected from 400 college students (56.3% women) from 16 to 63 years old. Fifteen trees were calculated in the training group for each sex, using different numbers and combinations of predictors. The result shows that for women BMI, WC, and WHR are the combination that produces the best prediction, since it has the lowest deviance (87.42), misclassification (.19), and the higher pseudo *R* ^2^ (.43). This model presented a sensitivity of 80.86% and specificity of 81.22% in the training set and, respectively, 45.65% and 65.15% in the test sample. For men BMI, WC, HC, and WHC showed the best prediction with the lowest deviance (57.25), misclassification (.16), and the higher pseudo *R* ^2^ (.46). This model had a sensitivity of 72% and specificity of 86.25% in the training set and, respectively, 58.38% and 69.70% in the test set. Finally, the result from the classification tree analysis was compared with traditional logistic regression, indicating that the former outperformed the latter in terms of predictive power.

## 1. Introduction

Obesity (body mass index > 29.9 kg/m^2^) has been considered a global public health problem due to its high prevalence and high morbidity [1]. In fact, the prevalence of obesity has increased substantially, both in developed and in under development countries. In the United States, for example, it is estimated that 35.5% of women and 32.2% of adult men present obesity [2]. The Brazilian Institute of Geography and Statistics (IBGE) indicates that 50.1% of men and 48% of women have overweight (25 kg/m^2^ ≤ BMI < 29.9 kg/m^2^), while 12.4% of men and 16.9% of women are suffering from obesity in Brazil [3].

The high risk attributed to obesity is related particularly to its association with increased risk factors for cardiovascular disease, notably hypertension [4, 5]. In order to adopt early preventive/therapeutic actions to minimize the risk of cardiovascular events in obese individuals, methods that can predict hypertension using low cost procedures are necessary, especially in underdeveloped and in developing countries.

Body mass index (BMI), waist circumference (WC), hip circumference (HC), and waist-hip ratio (WHR) are among the most practical and cost effective measures for evaluation of obesity, with the advantage that both WC and WHR present positive correlations with the amount of visceral fat, and together effectively predict cardiovascular risk [6, 7]. Furthermore, these anthropometric measures are predictors of metabolic factors and multiple health risks [8, 9]. Yong et al. [9] used the ROC curve analysis to verify the predictive power of WC, WHR, and BMI on blood pressure in 722 Chinese adults. WC presented a cutoff of 89.05 cm for men (sensibility = 70%, specificity = 42%, *P* < 0.001) and 90.90 cm for women (sensibility = 60%, specificity = 67%, *P* < 0.001). Waist-hip ratio was not a significant predictor for men (*P* = 0.369) or women (*P* = 0.070), with a cutoff of 0.92 cm for the first (sensibility = 67%, specificity = 54%) and 0.85 cm for the second (sensibility = 83%, specificity = 40%). Finally, BMI presented a cutoff of 23 kg/m^2^ for men (sensibility = 76%, specificity = 49%, *P* < 0.001) and 23.3 kg/m^2^ for women (sensibility = 75%, specificity = 59%, *P* < 0.001). Although less employed in the study of health conditions related to obesity, hip circumference is pointed as a variable that can increase the predictive power of the other anthropometric variables and should be included in the obesity studies [10]. It seems that the combination of multiple anthropometric variables increases the sensibility of the prediction [11, 12].

From the usual methods employed to study the relationship between anthropometric variables and obesity, the receiver-operating characteristic (ROC) curve analysis is the technique used to provide and to verify the quality of the cutoff points. This statistical method is highly recommended in epidemiological studies [13] because it can describe the accuracy of a variable to classify people into relevant clinical groups. However, the ROC curve methodology is not an informative technique to evaluate the contribution of an additional variable to the model [14]. The use of the ROC curve analysis became limited to investigate incremental validity, that is, the improvement in the prediction or in the amount of variance explained when an additional variable enters the model. Thus, in order to discover the strength of any combination of WC, HC, and WHR to predict hypertension, it is necessary to employ a statistical method that can provide sensitive information about incremental validity.

Health researches could benefit from employing machine learning techniques to verify the combination of variables that best predict a given outcome, as well as to verify their cutoff values. Machine learning is a relatively new science field focused on the construction and study of systems that can automatically learn from data [14], generating high accurate predictive models. Although incipient, machine learning methods are already in use in the health literature, as in the sustained weight loss study [15], in the evaluation of program cost effectiveness [16], in the obesity prediction [17], in the classification of prostate cancer levels [18], and in the classification of electronic patient records [19]. In 2013, The Microsoft Research Machine Learning Summit presented new applications of the machine learning techniques in health science, including applications to analyze clinical [20], genetic [21], and medical image data [22].

Among the techniques of machine learning, the *classification and regression tree* (CART) is of special interest for health studies, since it is useful: (1) to discover which variable, or combination of variables, better predicts a given outcome (e.g., presence of increased blood pressure,) and (2) to identify the cutoff values for each variable that maximally predicts the chosen outcome.

CART is a type of supervised learning technique [14] for recursively partitioning a feature space into several parts (or nodes), based on the relationship between an outcome variable and one or more predictors. The recursive binary partition is used to achieve a solution that divide the feature space into more pure nodes, that is, into a classification with the highest amount of cases with the same condition (e.g., hypertension). In sum, CART works as follows: (1) iteratively split variables into groups; (2) split the data where it is maximally predictive and (3) maximize the amount of homogeneity in each group [23].

Two main indexes, misclassification and deviance, can be used to indicate the quality of the prediction. Hastie et al. [14] explain how both work: In a node *m*, representing region *R*_*m*_, with *N*_*m*_ observations, let

(1) $$\begin{array}{c} {\hat{p}}_{mk}=\frac{1}{N_{m}}\sum_{x_{i}\in R_{m}}I\left(y_{i}=k\right) \end{array}$$

the proportion of class *k* observations in node *m*. We classify the observations in node *m* to class $(m)={\arg\;\max}_{k}\;{\hat{p}}_{mk}$, the majority class in node *m*. Different measures of node impurity (⋯) include the following:

Misclassification error: $(1/N_{m})\sum_{i\in R_{m}}I\left(y_{i}\neq k\left(m\right)\right)=1-{\hat{p}}_{mk}$.

Cross entropy or deviance: $-\sum_{k=1}^{K}{\hat{p}}_{mk}\log{\hat{p}}_{mk}$ (p. 309).

Misclassification is the index indicating the total amount of wrong predictions made or its rate (number of wrong predictions/total number of cases). Deviance is an index that is sensible to both the misclassification and the purity of the feature space partitions. As pointed by Hastie et al. [14] and by Golino et al. deviance is a better index to compare different models than misclassification, since it is more sensitive to node purity.

The present study has as the main goal to introduce and to apply the machine learning technique named classification and regression tree (CART) in the context of increased blood pressure. The machine learning field is a set of innovative techniques that provides state-of-the-art predictions in terms of accuracy. CART is becoming popular in different science fields since its interpretability is straightforward; the result of the prediction is easily understandable by experts of the field; it is applicable to a wide range of problems, can use any kind of variable as predictor, is a nonparametric technique, and is sensible to the impact of additional variables in the predictive model. Through the application of the CART analysis we expect to contribute with future studies focusing on the prediction of increased blood pressure by any kind of variable (e.g., genes, daily life habits, biomarkers, etc.). Additionally, we are going to compare the results from the CART analysis with traditional logistic regression analysis, in terms of strength of the prediction (pseudo-*R* ^2^ and AUC). In the present study we will analyze which variable, or combination of variables (BMI, WC, HC, and WHR), better predicts increased blood pressure (prehypertension or hypertension) and which cutoff values are maximally predictive of it. Fifteen models, or trees, with different number and combination of predictors will be compared for each sex, in a training sample. Then, the best tree will be tested in a testing sample for cross validation.

## 2. Materials and Methods

### 2.1. Sample and Measures

The data was collected in a convenience sample composed of 400 undergraduate students (56.3% women) aged between 16 and 63 years old (mean = 23.14 and standard deviation = 6.03), from a private university of Vitória da Conquista, Bahia, Brazil. All participants signed an informed consent agreeing to participate in the research. Weight was measured using a digital scale (Model B530, Plebal Plenna Ltda., SP, Brazil), to the closest 0.1 Kg. Height was measured using a stiff tape placed vertically on a flat wall, on subjects standing erect and head in the Frankfurt plane [24]. BMI was calculated using these measurements. WC was measured at the midpoint between the lower border of the rib cage and the iliac crest, and HC was measured at the greater gluteal curvature, both using a 1.5 meters' tape (ISP Eletromédica, Brazil), and recorded to the closest 0.1 cm. Blood pressure was measured using a manually inflatable blood pressure monitor (HEM-403INT, Omron Healthcare, Japan). All anthropometric measurements were repeated three times (the mean value was used in the data analysis) and were taken by previously trained research assistants.

### 2.2. Data Analysis

The systolic blood pressure was assessed and the subjects with increased blood pressure were identified. The data were first split into two subsets, one for each sex. Then, each subset was randomly split into two sets (training and testing) with almost the same number of people for cross-validation. The dataset is freely available in a web repository for reproducible purposes [25, 26]: (1) women's dataset can be downloaded at http://dx.doi.org/10.6084/m9.figshare.845664; (2) men's dataset can be downloaded at http://dx.doi.org/10.6084/m9.figshare.845665. All analyses were made using the *tree* package [27] from the R software. In the current study the *tree classification* procedure was fitted by binary recursive partitioning using as outcome the presence of increased blood pressure: at least prehypertension (systolic blood pressure > 120.0 mmHg) for women and hypertension (systolic blood pressure > 140.0 mmHg) for men. We are not investigating systolic hypertension in the weman sample because only 8% of them presented a systolic blood pressure equal to or greater than 140 mmHg. When the prevalence of one category of the outcome variable is very low, the classification tree fits a model that only predicts more abundant category. This problem is typical of the machine learning methods, which suffer in the presence of unbalanced datasets [28]. Geurts et al. [28] suggest to undersample the majority class in order to solve the problem, but we decided not to follow their advice, since our dataset contains only 18 women with systolic hypertension. To balance the data, by undersampling the majority class (no systolic hypertension), we would have created another issue: a very low sample size that would preclude the cross-validation. So, we decided to investigate prehypertension in the women sample, since 42% of them presented systolic blood pressure greater than 120 mmHg. The predictive variables included in the models were BMI, WC, HC, and WHR.

Fifteen random trees were calculated (grown) from the training set for each sex, in order to identify which variables, or which combination of variables, were suitable to predict the presence of increased blood pressure. Each random tree had one or more predictors, as can be seen in Table 2. The quality of each model or tree was verified using the misclassification error rate and deviance. A pseudo *R* ^2^ was calculated for each model, using the following formulae:

(2) $$\begin{array}{c} \text{Pseudo}\;R^{2}=1-\left(\frac{\text{Deviance}}{\text{SSY}}\right), \end{array}$$

where SSY represents the response sum of squares.

All the ethical principles contained in the Declaration of Helsinki were followed in the current study, as well as all the Brazilian specific laws.

## 3. Results and Discussion

None of the variables employed in the current study presented a normal distribution, as pointed by the Shapiro-Wilk's test of normality (see Table 1). Men presented a higher value of systolic blood pressure (median = 130 mmHg), BMI (median = 24 kg/m^2^), WC (median = 84 cm), HC (median = 103 cm), and WHR (median = 0.83) than women. The latter showed the following medians: a SBP of 117 mmHg, a BMI of 22 kg/m^2^, a WC of 76 cm, a HC of 100 cm, and a WHR of 0.76.

Systolic blood pressure presented a moderate correlation with all the anthropometric variables. Only 10.89% of SBP's variance was explained by BMI, 12.25% was explained by WC, 6.25% by HC, and 9.61% by WHR. Increased blood pressure was found in 42% of women (SBP > 120 mmHg) and in 47% of men (SBP > 140 mmHg). Table 2 shows the deviance, misclassification, and pseudo-*R* ^2^ for each of the fifteen models investigated in the training sample. Waist circumference alone was the worst predictor for the women sample (tree 2), since it presented a deviance of 149.30, a misclassification error rate of 0.40, and a pseudo-*R* ^2^ of only 0.03. Tree 13 presented the best model, with the lowest deviance (87.42), a misclassification error rate of 0.19, and a pseudo-*R* ^2^ of 0.43. Comparing the variables alone, body mass index was the best predictor, explaining 32% of the variance of increased systolic blood pressure for women, against only 3% for waist circumference and hip circumference and 9% of waist-hip ratio. When added to BMI as predictors, WC and HC worsen the prediction, decreasing the percentage of explained variance from 32% to 29% and the misclassification from 0.27 to 0.26 and increasing the deviance from 104.50 to 109.50 and 108.90, respectively. All combinations of three variables provided a better prediction than the variables alone or combined two by two. Tree 11, for example, had BMI, WC and HC as predictors of increased blood pressure and resulted in a better model then tree 1 (BMI alone), decreasing deviance from 104.50 to 94.24, misclassification from 0.26 to 0.22 and increasing the percentage of explained variance from 32% to 39%. So, WC and HC together with BMI have an incremental validity that adds 4% of explanation to the predictive model. However, the best model, represented by tree 13, showed that WC and WHR combined with BMI add 11% of variance explanation to the predictive model, compared to BMI alone (tree 1), decrease the percentage of misclassification from 26% to 19%, and decrease deviance from 104.50 to 87.42. So, WC and WHR have a considerable impact on the prediction of increased blood pressure in women when combined with BMI, presenting incremental validity over the latter.

Considering the men's result, waist-hip ratio alone (tree 4) presented the worst prediction model, with deviance of 95.09, misclassification error rate of 0.29, and pseudo-*R* ^2^ of 0.11. Tree 15 presented the best model, with the lowest deviance (57.25), a misclassification error rate of 0.16, and a pseudo-*R* ^2^ of 0.46. Comparing the variables alone, body mass index was the best predictor, explaining 16% of the variance of increased systolic blood pressure for men, with a deviance of 89.35 and a misclassification of 0.26, a result slightly better than tree 3 in which HC alone explained the same amount of increased SBP's variance with deviance of 89.59 and a misclassification of 0.26. When added to BMI as predictors, WC, HC, and WHR increase the percentage of variance explanation to 33%, 25%, and 25%, respectively, with deviance of 71.07, 80.17, and 79.90 and with misclassification error rate of 0.21, 0.25, and 0.23. All combinations of two variables lead to a better prediction than every variable alone. When added to BMI and WC, hip circumference worsens the prediction by decreasing the percentage of increased systolic blood pressure's variance explanation from 33% (tree 5) to 32% (tree 11), increasing deviance from 71.07 to 72.66, and misclassification error rate from 0.21 to 0.23. However, when waist-hip ratio is added to BMI, WC, and HC as predictors, deviance, misclassification, and pseudo-*R* ^2^ are improved. If we compare tree 15 with tree 1 it is possible to argue that WC, HC, and WHR together present incremental validity in the prediction of increased systolic blood pressure in men, increasing 30% of its variance explanation, improving 10% of misclassification, and decreasing deviance from 89.35 to 57.25.

**Figure 1:**
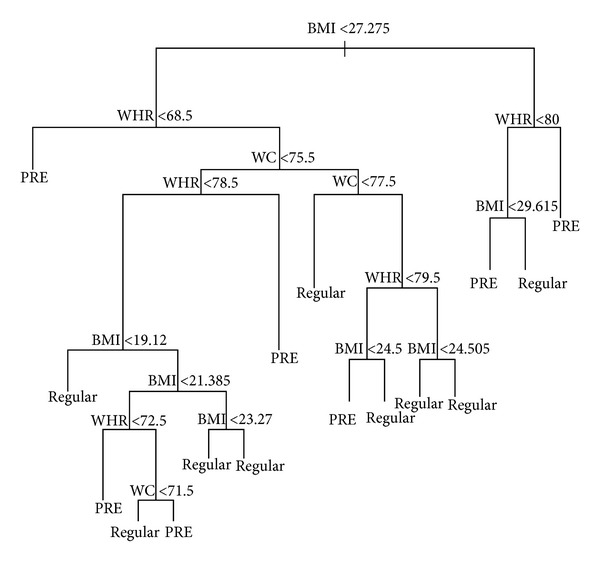
Best model for women. Notice that the endpoint for women is systolic blood pressure greater than 120 mmHg (prehypertension).

Figure 1 shows the tree that best predicts increased blood pressure for women (SBP > 120 mmHg) in the training sample. The predictors are distributed in several nodes and are always split in a specific cutoff value. The predictions made are at the bottom of the tree. For example, when BMI is smaller than 27.27 kg/m^2^ and WHR is smaller than .685 cm, the person is classified as having systolic pre-hypertension (classified as PRE). When BMI is higher than 27.27 kg/m^2^ and WHR is higher than 0.80, the person is also classified as having systolic pre-hypertension (classified as PRE). Averaging the percentage of women with increased blood pressure (or systolic pre-hypertension) classified as having pre-hypertension in each node results in a correct prediction of 80.86%; in other words this is the overall tree sensibility. In the same line of reasoning, averaging the percentage of women with regular blood pressure classified as having a regular pressure in each node results in a correct prediction of 81.22%; in other words this is the overall tree specificity. However, the cross-validation of tree 13 in women's testing sample showed that the sensibility decreased to 45.65% and the specificity to 65.15%. The percentage of women with SBP greater than 120 mmHg in the training sample was 43.75% and in the testing sample was 41.07%.

**Figure 2:**
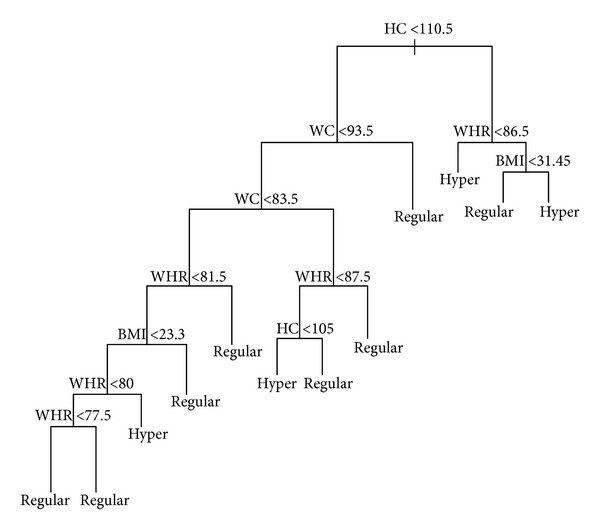
Best model for men. Notice that the endpoint for men is greater than 140 mmHg (hypertension).

Figure 2 shows the tree that best predicts increased blood pressure for men (SBP ≥ 140 mmHg) in the training sample. The interpretation of Figure 2 is the same as Figure 2. For example, when HC is higher than 110.5, WHR is higher than 0.865, and BMI is greater than 31.45, the person is classified as having systolic hypertension (HYPER). Actually, 67% of men with these characteristics have a systolic blood pressure greater than or equal to 140 mmHg. However, when HC is higher than 110.5, WHR is higher than 0.865, and BMI is smaller than 31.45, the person is classified as having regular systolic blood pressure (REGULAR). In fact, 80% of men with these characteristics have a systolic blood pressure lower than 140 mmHg. On one hand, averaging the percentage of men with increased blood pressure (or systolic hypertension) classified as having hypertension in each node results in a correct prediction of 72% (overall tree sensibility). On the other hand, averaging the percentage of men with regular blood pressure classified as having a regular pressure in each node results in a correct prediction of 86.25% (overall tree specificity). However, the cross-validation of tree 15 in men's testing sample showed that the sensibility decreased to 52.38% and the specificity to 69.70%. The percentage of men with SBP greater than or equal to 140 mmHg in the training sample was 30% and in the testing sample was 24.13%.

Comparing the strength of the predictions made, it is clear that classification trees outperformed traditional logistic regression. The best predictive model for women generated using classification trees had a pseudo-*R* ^2^ of 0.43, with an overall tree sensibility of 80.86% and specificity of 81.22%, while the logistic model with higher pseudo-*R* ^2^ and AUC was the model 11, presenting 0.023 and 0.566, respectively (see Table 3). The best classification tree for men presented a pseudo-*R* ^2^ of 0.466 with an overall sensibility of 72% and specificity of 86.25%, while the logistic model with higher pseudo-*R* ^2^ and AUC was the model 12, presenting 0.13 and 0.68 respectively (see Table 4).

## 4. Conclusions

According to the Harvard Obesity Prevention Source [29], it is estimated that 500 million adults are obese and 1.5 billion are overweight or obese worldwide. Obesity is a public health problem that affects approximately 1.5 million people each year in Brazil [30] and is responsible for a huge amount of money, about U$ 240 million dollars in 2011 [31], to directly treat it or to treat several related diseases. This chronic noncommunicable disease had its prevalence increased in both developed and in development countries, affecting, for example, 35.5% of women and 32.2% of adult men in the USA [2] and 12.4% of men and 16.9% of women in Brazil [3]. At least three pathophysiological mechanisms are known to link obesity to increased blood pressure. The first one is related to visceral obesity, indicating that mesenteric and omental adipocytes are more active than the subcutaneous ones [32], contributing to endothelial dysfunction, which may contribute to increasing blood pressure in obese people. The other two mechanisms involve the sympathetic nervous system [33, 34] and the imbalance in the homeostasis of plasma sodium [35–37] that are related to the extracellular volume increase and, thus, contribute to blood pressure elevation in people with obesity.

Anthropometric variables are among the most practical and low-cost obesity diagnostic methods [38], regarding their limitations and issues [38, 39]. World Health Organization 2008's report [40] points that body mass index, waist circumference, and waist-hip ratio are related to risk of cardiovascular diseases, hypertension, overall mortality, and other health problems. The same report points that additional information can be provided by hip circumference in the diagnosis of obesity, since it is related to gluteofemoral muscle mass and bone structure. Previous studies have pointed to the cutoff values of the anthropometric variables that are related to blood pressure [9, 41]. The cut-off values are different across ethnicities [40, 42], samples, age, and risk factors investigated [43]. All the studies quoted above employed traditional data analysis procedures, such as linear or logistic regression and the ROC curve analysis to verify the predictive role for each variable and to discover the best cutoff values for them. The use of null-hypothesis significance testing (*P* value) requires caution to verify which variables better predict obesity. A smaller *P* does not indicate a stronger relationship between independent and dependent variables, and statistical significance does not indicate practical importance [44]. The ROC curve analysis is highly recommended in epidemiological studies [45] because it can describe the accuracy of a variable to classify people into relevant clinical groups. However, the ROC curve methodology is not an informative technique to evaluate the contribution of an additional variable to the model [14], being limited to investigate the improvement in the prediction or in the amount of variance explained when an additional variable enters the model (incremental validity). In order to identify the role of BMI, WC, WHR, and HC together in the prediction of increased blood pressure it is necessary to employ a statistical method that can provide sensitive information about incremental validity.

A conjoint of techniques that can provide sensitive information about incremental validity is the *classification and regression tree* (CART) of the machine learning field. The CART techniques are of special interest for health studies to discover which variable, or combination of variables, better predicts a given outcome (e.g., presence of increased blood pressure) and to identify the cutoff values for each variable that maximally predicts the chosen outcome. Health studies have been employing machine learning methods in different applications that go from sustained weight loss investigation [46], obesity prediction [17], to genetic [21] and medical image data analysis [22].

In the current study, classification tree models were used to verify which combination of BMI, WC, WHR, and HC best predicted increased blood pressure for women and men. The best model for women showed that adding WC and WHR to BMI increased 11% of the variance explanation in the predictive model, decreased the percentage of misclassification from 26% to 19%, and decreased deviance from 104.50 to 87.42. As pointed before, WC and WHR have a considerable impact on the prediction of increased blood pressure in women when combined with BMI. The overall sensibility for the best model (tree no. 13) was 80.86% and the overall specificity was 81.22%. The complex model represented by tree 13 (see Figure 1) exceeded the sensitivity and specificity found in previous studies. Yong et al. [9] found sensibility and specificity of 60% and 67% for WC, 83% and 40% for WHR, and 75% and 59% for BMI in the women sample. A Brazilian study investigating the predictive role of BMI in hypertension in 1,298 people (52.5% of women) found an area under the ROC curve of 0.69 (95% C.I.: 0.64–0.74) for women [47].

The best model for men showed that WC, HC, WHR, and BMI together presented an incremental validity over BMI alone in the prediction of hypertension, increasing 30% of the variance explanation, improving 10% of misclassification, and decreasing deviance from 89.35 to 57.25. The overall sensibility and specificity for the best model (tree no. 15) was 72% and 86.25%. As happened with the women result, the complex model represented by tree 15 (see Figure 2) exceeded the sensitivity and specificity found in previous studies. Furthermore, compared to traditional logistic regression analysis, the classification trees produced a much better prediction, with higher pseudo-*R* ^2^, sensibility, and specificity for both men and women.

In spite of the high sensibility and specificity of the best models for both men and women in the training sample, the cross validation applied in the test sample revealed a different scenario. The cross-validation of tree 13 in women's testing sample showed that the sensibility decreased to 45.65% and the specificity to 65.15%, and the cross-validation of tree 15 in the men's testing sample showed that sensibility decreased to 52.38% and the specificity decreased to 69.70%. The observed difference in sensibility and specificity between the modeled training set and the cross-validation in the test set is known in the machine learning literature as the *variance* issue [28]. It means that the algorithm learned too much from the observed data and is likely to make more errors in a different data set. So, we need to interpret the present result and the best model for both men and women with caution. The variance issue that emerged from our study can also emerge in studies using more traditional statistical methods, such as the ROC curve analysis. It is important to make subset of the sample gathered in order to apply cross-validation. The result found by Abolfotouh and colleagues [8], or by every other investigation made that did not use a cross-validation procedure, may be susceptible of the variance issue. However, overfitting is more usual in the machine learning techniques than in the traditional ones. In order to overcome overfitting in the classification and regression tree's method, a bootstrap procedure named Random Forests [48] can be applied. It bootstraps samples and variables, grows multiple trees, enables greater accuracy, and avoids overfitting, being one of the best procedures for dealing with the variance issue [28]. Finally, we must address the limitations of the current study. Firstly, it did not employ a representative sample randomly chosen, relying on a convenience sample. It makes our inferences limited. Secondly, the number of women with hypertension was very low, obligating us to analyze increased blood pressure in them and hypertension in men, which compromised the comparison of the findings between sexes. Those issues should be solved in future researches. Finally, our results cannot be generalized to other ethnics, but the methodology adopted in the current paper could be used in data gathered in different countries to construct new predictive models of increased blood pressure.

Using machine learning techniques to discover new relations in data, to verify incremental validity of additional predictors, and to make accurate predictions for new data sets may help the health scientists to find new robust diagnostic parameters. The clinical usefulness of the present study relies on the possibility of using new algorithms to classify and predict increased blood pressure, with higher accuracy than usual cutoff points. Although most of the clinicians can measure both blood pressure and the anthropometric variables simultaneously, there are several parts of the world, such as many countries from Africa or several places in Latin America, where material resources are scarce. So, the application of complex algorithms, as the presented one in the current paper, can be a help for those professionals that can rely only on very simple and cheap instruments, such as a measure tape. Furthermore, the present study applied a new method for prediction of health outcomes, which in spite of being incipient in the literature, can provide new insights and discoveries since it outperforms traditional techniques (such as logistic and linear regression), making possible to compare the impact of new variables on the prediction of the chosen outcome (incremental validity). Traditional techniques are based on several assumptions, as the normality of the distribution, linear relationship between independent and dependent variables, homoscedasticity, and so on. The Machine learning techniques can handle any kind of variable (ordinal, continuous, dichotomous, and nominal), with no assumption about distribution, linearity, or homoscedasticity. Moreover, it can be used to extract useful information and to discover new relations in very huge data sets provided by some international databases, such as the World Health Organization Global database on noncommunicable diseases (see http://www.who.int/gho/ncd/en/index.html) [49]. As quoted in the introduction, the data deluge can transform the society, and machine learning will play a pivotal role in it. Future researches should overcome the limitations of the present study by employing a larger and representative sample, by using strategies to minimize the variance issue, especially the Random Forest [48] approach.

## Figures and Tables

**Table 1:** Descriptive statistics for SBP, BMI, WC, HC, and WHR; prevalence of obesity for woman and man; test of normality and correlations.

	Woman				Man				Normality test		Spearman's correlation			
	Mean	SD	Median	*N* (%)	Mean	SD	Median	*N* (%)	Shapiro-Wilk's W	SBP	BMI	WC	HC	WHR
SBP	118.66	15.91	117.00		132.35	14.44	130.00		0.96***	1	0.33*	0.35*	0.25*	0.31*
BMI	22.76	4.08	22.00	225 (56.3%)	24.46	4.34	24.00	175 (43.8%)	0.93***	0.33*	1	0.80*	0.81*	0.46*
WC	76.95	9.47	76.00		86.06	11.47	84.00		0.95***	0.35*	0.80*	1	0.73*	0.77*
HC	100.71	8.79	100.00		102.93	8.93	103.00		0.98**	0.25*	0.81*	0.73*	1	0.20*
WHR	0.76	0.07	0.76		0.83	0.07	0.83		0.94***	0.31*	0.46*	0.77*	0.20*	1
SBP > 120 mmHg														
No				130 (58%)		SBP > 140 mmHg		128 (73%)						
Yes				95 (42%)				47 (39%)						

*Significant at the 0.01 level.

**Significant at the 0.05 level.

***Significant at the 0.001 level.

SBP: systolic blood pressure, BMI: body mass index, WC: waist circumference, HC: hip circumference, WHR: waist-hip ratio. Notice that the endpoint for women is SBP greater than 120 mmHg (prehypertension), while for men is SBP greater than 140 mmHg (hypertension).

**Table 2:** Predictors, deviance, misclassification, and pseudo-*R* ^2^ by tree.

Tree	Predictors	Women			Men		
		Deviance	Misclassification error rate	Pseudo- *R* ^2^	Deviance	Misclassification error rate	Pseudo- *R* ^2^
1	BMI	104.50	0.27	0.32	89.35	0.26	0.16
2	WC	149.30	0.40	0.03	92.96	0.27	0.13
3	HC	148.90	0.41	0.03	89.59	0.26	0.16
4	WHR	139.50	0.33	0.09	95.09	0.29	0.11
5	BMI + WC	109.50	0.26	0.29	71.07	0.21	0.33
6	BMI + HC	108.10	0.26	0.29	80.17	0.25	0.25
7	BMI + WHR	104.60	0.23	0.32	79.9	0.23	0.25
8	WC + HC	149.30*	0.40	0.03	82.19	0.23	0.23
9	WC + WHR	115.90	0.26	0.24	69.54	0.24	0.35
10	HC + WHR	118.60	0.29	0.23	76	0.21	0.29
11	BMI + WC + HC	94.24	0.22	0.39	72.66	0.23	0.32
12	WC + HC + WHR	99.17	0.23	0.35	61.5	0.17	0.42
13	BMI + WC + WHR	87.42	0.19	0.43	64.98	0.19	0.39
14	BMI + HC + WHR	101.10	0.22	0.34	61.93	0.16	0.42
15	BMI+ WC + HC + WHR	89.46	0.19	0.42	57.25	0.16	0.46

*HC dropped out.

Notice that the endpoint for women is systolic blood pressure greater than 120 mmHg (prehypertension), while for men is greater than 140 mmHg (hypertension).

**Table 3:** Logistic regression results of women's training set.

	Coefficients	S.E	Wald *Z*	*P*	Pseudo*R* ^2^	AUC
Model 1						
Intercept	17.615	12.206	1.44	0.1490	0.019	0.548
BMI	−0.0651	0.0519	−1.25	0.2095		
Model 2						
Intercept	20.584	17.149	1.20	0.2300	0.014	0.529
WC	−0.0236	0.0223	−1.06	0.2886		
Model 3						
Intercept	18.701	22.687	0.82	0.4098	0.006	0.542
HC	−0.0160	0.0224	−0.72	0.4737		
Model 4						
Intercept	25.470	29.026	0.88	0.3802	0.008	0.539
WHR	−0.0303	0.0382	−0.79	0.4278		
Model 5						
Intercept	17.497	17.739	0.99	0.3240	0.019	0.549
BMI	−0.0659	0.0973	−0.68	0.4987		
WC	0.0004	0.0418	0.01	0.9927		
Model 6						
Intercept	0.5958	25.251	0.24	0.8135	0.022	0.572
BMI	−0.1030	0.0891	−1.16	0.2477		
HC	0.0203	0.0386	0.53	0.5993		
Model 7						
Intercept	26.319	29.105	0.90	0.3658	0.020	0.541
BMI	−0.0579	0.0562	−1.03	0.3025		
WHR	−0.0137	0.0414	−0.33	0.7414		
Model 8						
Intercept	16.879	22.829	0.74	0.4597	0.014	0.534
WC	−0.0311	0.0378	−0.82	0.4103		
HC	0.0093	0.0381	0.25	0.8063		
Model 9						
Intercept	23.660	29.157	0.81	0.4171	0.014	0.529
WC	−0.0211	0.0293	−0.72	0.4711		
WHR	−0.0066	0.0503	−0.13	0.8961		
Model 10						
Intercept	38.639	35.464	1.09	0.2759	0.013	0.529
HC	−0.0146	0.0225	−0.65	0.5151		
WHR	−0.0282	0.0383	−0.73	0.4626		
Model 11						
Intercept	0.6938	25.681	0.27	0.7870	0.023	0.566
BMI	−0.0904	0.1069	−0.85	0.3976		
WC	−0.0097	0.0454	−0.21	0.8317		
HC	0.0237	0.0419	0.57	0.5711		
Model 12						
Intercept	−235.909	29.736	6−0.79	0.427	0.023	0.539
WC	−0.3548	0.382	1−0.93	0.353		
HC	0.2571	0.293	5 0.88	0.381		
WHR	0.3303	0.387	5 0.85	0.394		
Model 13						
Intercept	28.202	29.786	0.95	0.3437	0.021	0.558
BMI	−0.0856	0.1070	−0.80	0.4239		
WC	0.0170	0.0559	0.30	0.7609		
WHR	−0.0249	0.0554	−0.45	0.6538		
Model 14						
Intercept	10.603	46.901	0.23	0.8211	0.022	0.566
BMI	−0.0962	0.1061	−0.91	0.3646		
HC	0.0181	0.0426	0.43	0.6699		
WHR	−0.0054	0.0458	−0.12	0.9064		
Model 15						
Intercept	−22.3777	29.6673	−0.75	0.4507	0.030	0.57
BMI	−0.0833	0.1078	−0.77	0.4399		
WC	−0.3078	0.3851	−0.80	0.4242		
HC	0.2495	0.2926	0.85	0.3939		
WHR	0.3025	0.3876	0.78	0.4351		

Notice that the endpoint for women is systolic blood pressure greater than 120 mmHg (prehypertension), while for men is greater than 140 mmHg (hypertension).

**Table 4:** Logistic regression results of men's training set.

	Coefficients	S.E	Wald *Z*	*P*	Pseudo *R* ^2^	AUC
Model 1						
Intercept	3.27	1.41	2.32	0.02	0.05	0.597
BMI	−0.09	0.05	−1.74	0.08		
Model 2						
Intercept	3.28	1.73	1.89	0.05	0.03	0.591
WC	−0.02	0.01	−1.42	0.15		
Model 3						
Intercept	7.99	3.05	2.62	0.008	0.09	0.656
HC	−0.06	0.02	−2.36	0.0183		
Model 4						
Intercept	0.12	3.15	0.04	0.96	0.001	0.498
WHR	0.0089	0.03	0.24	0.8120		
Model 5						
Intercept	28.040	17.829	1.57	0.1158	0.05	0.596
BMI	−0.1416	0.1222	−1.16	0.2463		
Wc	0.0193	0.0442	0.44	0.6628		
Model 6						
Intercept	106.641	42.606	2.50	0.0123	0.10	0.667
BMI	0.1117	0.1202	0.93	0.3528		
HC	−0.1213	0.0645	−1.88	0.0601		
Model 7						
Intercept	−15.975	33.454	−0.48	0.6330	0.09	0.636
BMI	−0.1641	0.0710	−2.31	0.0207		
WHR	0.0797	0.0499	1.60	0.1105		
Model 8						
Intercept	101.735	35.689	2.85	0.0044	0.12	0.672
WC	0.0532	0.0404	1.32	0.1883		
HC	−0.1339	0.0587	−2.28	0.0226		
Model 9						
Intercept	−49.908	38.546	−1.29	0.1954	0.13	0.677
WC	−0.1085	0.0399	−2.72	0.0065		
WHR	0.1836	0.0770	2.38	0.0172		
Model 10						
Intercept	49.970	36.852	1.36	0.1751	0.12	0.678
HC	−0.0904	0.0339	−2.67	0.0077		
WHR	0.0633	0.0451	1.40	0.1602		
Model 11						
Intercept	106.368	42.926	2.48	0.0132	0.12	0.671
BMI	0.0284	0.1449	0.20	0.8447		
WC	0.0479	0.0485	0.99	0.3234		
HC	−0.1408	0.0686	−2.05	0.0400		
Model 12						
Intercept	−208.656	32.535	2−0.6	4 0.521	0.13	0.680
WC	−0.2790	0.350	1−0.8	0 0.425		
HC	0.1460	0.296	7 0.4	9 0.622		
WHR	0.3697	0.387	9 0.9	5 0.340		
Model 13						
Intercept	−55.980	41.837	−1.34	0.1809	0.13	0.681
BMI	0.0537	0.1476	0.36	0.7161		
WC	−0.1345	0.0820	−1.64	0.1013		
WHR	0.2015	0.0916	2.20	0.0279		
Model 14						
Intercept	57.797	60.170	0.96	0.3368	0.12	0.679
BMI	0.0234	0.1420	0.16	0.8691		
HC	−0.0999	0.0671	−1.49	0.1364		
WHR	0.0586	0.0530	1.11	0.2687		
Model 15						
Intercept	−22.9141	32.9873	−0.69	0.4873	0.139	0.688
BMI	0.0614	0.1482	0.41	0.6787		
WC	−0.3231	0.3669	−0.88	0.3785		
HC	0.1582	0.2985	0.53	0.5960		
WHR	0.4062	0.3993	1.02	0.3089		
